# Entry Into Boarding Preschool Is Associated With Increased Stress and School Refusal

**DOI:** 10.1002/smi.70022

**Published:** 2025-03-05

**Authors:** Nan Xiao, Xiao Zhang, Cecilia Lai Wan Chan

**Affiliations:** ^1^ T. Denny Sanford Harmony Institute Arizona State University Tempe Arizona USA; ^2^ Faculty of Education The University of Hong Kong Hong Kong China; ^3^ Department of Social Work and Social Administration The University of Hong Kong Hong Kong China

**Keywords:** alpha‐amylase, boarding preschool, cortisol, physiological stress markers, school refusal

## Abstract

In western China, many socioeconomically disadvantaged rural children remain at preschools for extremely long hours: they start to receive boarding services as young as 3 years old and remain at preschools for 4 to 5 consecutive days weekly. Although the quality of childcare is generally poor in these preschools, extremely long school hours and prolonged separation from primary caregivers may induce additional stress and lead to school maladjustment among boarding preschoolers. This study examines the impact of boarding experience on preschool beginners' social adjustment and stress responses, as indicated by their saliva alpha‐amylase (sAA) and cortisol. A total of over 900 saliva samples of 31 boarding and 30 non‐boarding preschoolers (mean age = 44.0 months, SD = 9.8 months) were collected over 12 weeks after they entered preschools. Primary caregivers reported children's behavioural problems and school refusal. Boarders experienced a larger mid‐morning to mid‐afternoon rise in cortisol than non‐boarders on the second‐to‐last weekdays but not on the first weekdays. Non‐boarders experienced an accelerated decrease in sAA during the 12 weeks, whereas boarders did not. Differences in cortisol and sAA patterns indicate potential increased stress for boarders, which might be associated with their more prevalent school‐refusal behaviour than non‐boarders. The findings underscore that entry into boarding preschool may cause stress and school refusal in rural Chinese children during their transition to preschool. Additional contacts with primary caregivers during this transition are needed to support boarders emotionally.

## 1 Introduction

Boarding during preschool, where young children stay at preschool throughout the weekdays and only return home on weekends (or even less frequently), is a major source of stress for socioeconomically disadvantaged children in rural areas of China. In the literature, socioeconomically disadvantaged young children are disproportionately affected by stress (Eckstein‐Madry, Piskernik, and Ahnert 2021). For boarders, in particular, group care and prolonged separation from primary caregivers can also induce stress (Vermeer and van IJzendoorn 2006). In addition, although high‐quality childcare can protect children from stress (Hatfield et al. 2013), the quality of rural Chinese preschools is usually less than satisfactory (Sun and Li 2013) and likely adds additional stress to rural boarding preschoolers. Such heightened stress could impoverish children's immune functioning (Watamura et al. 2010) and impede flexible regulation of stress physiology in the long run (Ali and Pruessner 2012). Stress is also associated with behavioural adjustment at school, such as school refusal (Gonzálvez et al. 2018). Although boarding is a significant stressor, it is an unavoidable situation for many socioeconomically disadvantaged rural young children to access preschool education. Boarding is needed potentially due to long travel distances from home to school and parents working elsewhere as migrant workers. According to a survey covering 260 rural preschools in China's Yunnan province, 32% of the preschools provided boarding services, and some boarders were as young as 3 years old (Sun and Li 2013). In Chinese culture, boarding at a young age is an acceptable practice, and educators perceive it as a valuable service supporting parents and the family (Tobin, Wu, and Davidson 1989; Tobin, Hsueh, and Karasawa 2009). According to ethnographic work done by Tobin, Hsueh, and Karasawa (2009), parents perceive boarding as an opportunity for training children's self‐care skills, self‐discipline and independence. However, there is a lack of empirical evidence on the implication of boarding at a young age for children's adjustment. Based on a sample of rural Chinese children who were navigating their transition from home to preschool, this study examined the impact of boarding experience on their stress responses—as indexed by salivary cortisol and alpha‐amylase (sAA)—and social adjustment, as indicated by problem behaviour and school refusal.

Both salivary cortisol and Enzyme *α*‐amylase (sAA) are useful biological stress indicators. The cortisol level follows a normal diurnal rhythm that decreases from mid‐morning to mid‐afternoon (Vermeer and van IJzendoorn 2006). More recently, sAA has been utilised as a physiological marker for stress‐included sympathetic nervous system activities (Rohleder and Nater 2009), and momentary sAA is closely related to overall emotional arousal (Hatfield et al. 2013). Because cortisol and sAA reflect different stress response components, including both indicators in this study will provide a better understanding of the stress responses among these preschoolers.

*Attachment Theory* (Bowlby 1965) and *Human Ecology Theory* (Bronfenbrenner 1979) help explain why boarding is associated with stress. Boarding is a type of repeated short‐period maternal deprivation (e.g., at least 5 days per week). Early maternal deprivation is stressful for young children, according to Attachment Theory (Ahnert et al. 2004), and such early adversity can make children more emotionally sensitive (Bowlby 1980). Changes in cortisol can capture the increase in stress induced by maternal separation (Gunnar and Donzella 2002). Second, according to Human Ecology Theory, transferring from home to the childcare environment is a process of adapting to changes in the microsystem (e.g., interaction with new peers and teachers). The dramatic change is more challenging for boarders than non‐boarders since the former have less chance to spend with primary caregivers (e.g., parents) in the home environment (the original microsystem), while interactions with primary caregivers can help young children restore emotional equilibrium from the stress induced by childcare as indicated by biological indicators such as cortisol (Ahnert and Lamb 2003).

Preschoolers generally show an increasing or flattened pattern in morning‐to‐afternoon cortisol level changes among those in childcare compared to a reducing pattern among those at home (Vermeer and van IJzendoorn 2006). Longitudinal studies on children's transition to childcare found that children produced more total cortisol (Yang et al. 2017) or a larger morning‐to‐afternoon cortisol rise (Bernard et al. 2015) upon entry into childcare than those at home. There could be multiple explanations for the rise in cortisol levels. Firstly, separation from the primary caregiver is stressful, especially for insecurely attached children (Ahnert et al. 2004). Secondly, early institutional care experience has a sustained impact on hypothalamic–pituitary–adrenal axis functioning (Gunnar et al. 2010). Other explanations concern contextual factors in childcare settings, such as stressful peer interactions (Bernard et al. 2015). Moreover, additional hours spent in care were associated with a greater morning‐to‐afternoon cortisol rise (Drugli et al. 2017; Lumian et al. 2016). Because boarding can be regarded as a more extreme case than long hours in daycare, boarders may experience a larger morning‐to‐afternoon rise in cortisol at preschool than non‐boarders.

Little is known about the implications of childcare for preschoolers' sAA, which is related to emotional arousal (Hatfield et al. 2013). Because a lack of emotional support from primary caregivers could induce greater emotional instability or arousal among boarders (Ahnert and Lamb 2003), boarders might produce a higher level of sAA than non‐boarders. Moreover, according to Sun and Li (2013), teachers at rural boarding preschools provide limited emotional support to children, so teachers' emotional support may not compensate for boarders' loss of emotional support from caregivers. Hatfield et al. (2013) found that lower classroom emotional support predicted higher total daily sAA but not the diurnal change.

Although boarding may be stressful, it is unclear whether such stress would influence children's social adjustment. Furthermore, attending residential childcare (e.g., orphanages or children's homes) may be associated with behavioural problems during preschool years (Lee et al. 2010). For school‐age children, boarders were also disadvantaged in internalising and externalising problems compared to non‐boarding students (Ak and Sayil 2006). No research evidence was noted on children's behavioural problems during their transition to boarding preschools. Nevertheless, boarding preschoolers share ecological risk factors with school‐age borders, such as lack of emotional support, restricted physical living space, and isolation from their neighbourhood community.

School refusal is another behavioural indicator of social adjustment. School refusal (i.e., children's refusal to remain in class) leads to family conflicts and academic problems (Kearney, Chapman, and Cook 2005). Kearney et al. also found that school refusal behaviour was characterised by separation anxiety and attention‐seeking motives. Although no relevant research evidence was noted for boarding preschoolers, it is worth noting that boarders may lack emotional support from primary caregivers, thereby craving adult attention and experiencing separation anxiety. Moreover, with limited emotional support, boarders may find it challenging to deal with negative emotions. Thus, some motivations behind school refusal behaviour, such as avoiding stimuli provoking negative affectivity (e.g., crying) and pursuit of attention from caregivers, may be relevant in the boarding preschool context.

Rural boarding preschools in China are distinguishable from boarding schools commonly known in developed countries such as the United Kingdom, Australia, Canada and the United States (Martin et al. 2016) because of their under‐resourced nature. They are also special compared to other rural Chinese boarding schools since they serve very young children and are often left out of discussion as part of the system (e.g., Zhao 2011). However, these schools are worth special attention from the perspective of cumulative risk (Sameroff 2000), such that the co‐occurrence of risk factors for child development can lead to greater developmental disadvantages beyond the effect of individual factors. Empirical research has found support for the cumulative risk hypothesis both in terms of child physiological stress (Evans and Kim 2007) and behaviour outcomes (Appleyard et al. 2005). Rural Chinese preschoolers are subject to multiple risk factors from both home (e.g., poverty, parents working elsewhere) and childcare settings, such as lack of teach‐child closeness, low‐quality peer interaction and lack of free‐play time (Sun and Li 2013; Xiao et al. 2023). Boarding experience, on top of all these stressors, can induce additional burdens on these young children's physiological, psychological and behavioural well‐being and weaken their social support system by limiting parent‐child interactions. Thus, the present study focuses on a population who are in bad need of additional support and intervention efforts.

The present study examines boarders' stress responses over time and their social adjustment (problem behaviour and school refusal behaviour) compared to non‐boarders within the same school contexts. Based on past evidence (e.g., Hatfield et al. 2013), contextual factors might make a difference in diurnal cortisol patterns and daily sAA but not in daily cortisol or diurnal sAA patterns. Therefore, this study focuses on diurnal cortisol patterns and daily sAA levels. Three hypotheses were generated. First, boarders would experience a greater morning‐to‐afternoon cortisol rise [H1‐1] and a higher daily sAA level [H1‐2] than non‐boarders during the transition to preschool. Second, the boarding experience moderates the change trajectory of cortisol rise [H2‐1] and daily sAA level [H2‐2]. Third, boarders would exhibit more social adjustment problems (i.e., school refusal and behavioural problems) than non‐boarders [H3].

## 2 Method

### 2.1 Participants

A total of 61 preschool beginners (29 girls and 32 boys) with an average age of 44.0 months (SD = 9.8) and their primary caregivers were recruited from six rural boarding preschools in one of the poorest counties in Yunnan Province, China (For school characteristics, refer to Table 1). Children were identified before entering preschool based on the school registration system, and they had not attended preschools before participating in this study. The children included 31 boarders and 30 non‐boarders from the same six preschools (for distribution of boarders and non‐boarders, see Table 2), all from families of low socioeconomic status. Most parents were *farmers, non‐technical workers, or unemployed* (93% of fathers and 92% of mothers fell into this category) and did not attend high school (72% of fathers and 68% of mothers). Three children (< 5%) dropped out during the 12‐week follow‐up period due to primary caregivers' wishes (*n* = 2) and expulsion (*n* = 1). For details, refer to Table 3.

**TABLE 1:** Characteristics of the selected schools.

	Sample average	Minimum	Maximum
Student enrolment	106.0	51	148
Boarding rate	50%	14%	97%
Number of teachers/nurses	8.3	4	13
Number of other staff	2.8	1	4
Number of classes	4.3	3	7
Pre‐primary	0.3	0	2
Senior	1.7	1	2
Middle	1.5	1	2
Junior	0.7	0	1
Infant and Toddler	0.3	0	1
Mixed Age	0.0	0	0
Child‐to‐teacher ratio	13:1	10:1	26:1

Teacher education	Percentage
Junior middle school or below	16%
High school	8%
Vocational high school	6%
Secondary normal (technical secondary) school	10%
Advanced vocational college	24%
Associate's degree	30%
Degree	6%
Master's or above	0%

	Percentage of teachers	
	Yes	No
Possesses a teacher qualification certificate	46%	54%

	Percentage of schools	
	Private	Public
Privately/Publicly owned	83%	17%

*Note: N* = 6. Rural preschools commonly do not distinguish teachers and nurses very strictly. Some classes have only one adult in charge, so the teacher may also take on the role of nurse. Other staff positions include support staff such as chef, driver, and security guard.

**TABLE 2:** Distribution of boarders and non‐boarders across classrooms.

	Total	Boarders	Non‐boarders
Preschool 1	*n* = 7	*n* = 6	*n* = 1
Preschool 2	*n* = 8	*n* = 1	*n* = 7
Preschool 3	*n* = 10	*n* = 5	*n* = 5
Preschool 4	*n* = 7	*n* = 5	*n* = 2
Preschool 5	*n* = 16	*n* = 10	*n* = 6
Preschool 6	*n* = 13	*n* = 4	*n* = 9

*Note:* The participants were not evenly distributed across preschools, mainly due to the availability of newly enroled students who were willing to consent.

**TABLE 3:** Demographics of the sample participants.

Variable	Boarders		Non‐boarders	
	*M* (SD) %		*M* (SD) %	
Child characteristics	(*n* = 31)		(*n* = 30)	
Age (months)	48.4 (9.4)		39.4 (8.1)	
Sex (numeric code)	(*n* = 31)		(*n* = 30)	
Boy (1)	48%		57%	
Girl (0)	52%		43%	
Left behind status (numeric code)	(*n* = 31)		(*n* = 29)	
Yes (1)	26%		41%	
No (0)	74%		59%	
Family characteristics				
Annual income in 2019 (numeric code)	Mothers (*n* = 31)	Fathers (*n* = 29)	Mothers (*n* = 28)	Fathers (*n* = 27)
< 2000 RMB or $307 (1)	39%	7%	29%	4%
2000 ∼ 5000 RMB or $768 (2)	16%	14%	7%	4%
5000 ∼ 10,000 RMB or $1536 (3)	26%	21%	11%	7%
10,000 ∼ 20,000 RMB or $3073 (4)	16%	28%	11%	22%
20,000 ∼ 30,000 RMB or $4609 (5)	3%	17%	14%	19%
30,000 ∼ 50,000 RMB or $7682 (6)	0%	7%	11%	30%
50,000 ∼ 80,000 RMB or $12,291 (7)	0%	7%	7%	7%
80,000 ∼ 100,000 RMB or $15,363 (8)	0%	0%	7%	7%
≥ 100,000 RMB (9)	0%	0%	4%	0%
Parental education (numeric code)	Mothers (*n* = 31)	Fathers (*n* = 29)	Mothers (*n* = 29)	Fathers (*n* = 28)
Elementary school or below (1)	23%	24%	10%	7%
Middle school (2)	55%	59%	48%	54%
High school or vocational school degree (3)	23%	17%	21%	29%
Associate's degree (4)	0%	0%	7%	7%
Bachelor's degree or above (5)	0%	0%	14%	4%
Parental occupation (numeric code)	Mothers (*n* = 30)	Fathers (*n* = 28)	Mothers (*n* = 30)	Fathers (*n* = 30)
Unemployed, nontechnical worker, farmer (1)	100%	100%	83%	87%
Semitechnical worker, self‐employed small business owner (e.g., construction worker) (2)	0%	0%	7%	7%
Technical worker or semi‐professional (e.g., driver) (3)	0%	0%	0%	3%
Professional or officer (e.g., doctor, teacher, technician) (4)	0%	0%	10%	3%
High‐level professional or administrator (e.g., manager) (5)	0%	0%	0%	0%

### 2.2 Ethical Considerations

This study was approved by the Human Research Ethics Committee at the University of Hong Kong (EA1711002). Written informed consent was obtained from the caregivers, teachers, and principals of the children's preschools, and oral consent was obtained from the children. Research assistants, who were education major students from a local college, were given standardised protocol and training on how to conduct the sample collection in a warm, considerate manner and instructed to stop the collection when children displayed unwillingness to continue.

### 2.3 Procedure and Measures

Before children started preschool, their primary caregivers reported on demographics (child age, sex, family SES, ethnic minority and left‐behind child status) and behavioural problems of the children. Caregivers also reported school refusal and behavioural problems of children in Week 12. Saliva samples were collected in mid‐morning and mid‐afternoon on the first weekdays and second‐to‐last weekdays of Weeks 1, 2, 4, 8, and 12 upon entry into preschool to capture the trend in the diurnal rhythm of cortisol and sAA. Although an assessment of diurnal rhythm typically includes more daily samples, such as in the early morning and before bed (e.g., Tervahartiala et al. 2021), collecting two samples in the morning and afternoon captures the trend of diurnal rhythm as decreasing, flattening or elevating, which was widely adopted in previous studies (e.g., Bernard et al. 2015; Lumian et al. 2016). Moreover, it was not practical to collect and store early‐morning and before‐sleep samples from non‐boarders in rural settings. The second‐to‐last weekday, instead of the last weekday, was selected because caregivers usually picked up children around noon on the last weekday. Because some children were late on the first day of school, Tuesday samples were also collected in the first week as a supplement, adding up to 11 days per child. It was instructed that morning samples should be collected around 9:30 AM and afternoon samples around 4:00 PM. The mean sampling time fell within 40 min of the designated time. For details of collection times, please refer to Table 4.

**TABLE 4:** Descriptive statistics for cortisol and sAA value.

Week no.	Weekday	Collection session	Collection time			Cortisol					sAA			
						Raw (ng/mL)	Log‐transformed	*M* (SD) of LDC			Raw (IU/mL)	*M* (SD) of AUC		
			*M* (SD)	Min.	Max.	*M* (SD)	*M* (SD)	Whole sample	Boarders	Non‐boarders	*M* (SD)	Whole sample	Boarders	Non‐boarders
1	1st	AM	10:08 (00:41)	9:06	12:14	12.48 (28.96)	−0.44 (3.57)	−0.23 (1.59)	−0.58 (1.49)	0.23 (1.66)	260.18 (239.92)	46.68 (29.23)	47.79 (34.09)	45.39 (23.76)
		PM	15:43 (00:34)	14:50	16:51	15.04 (30.44)	0.02 (1.40)				183.89 (122.34)			
	2nd	AM	9:59 (00:40)	8:59	10:54	5.75 (22.69)	−0.85 (2.02)	0.67 (2.34)	0.80 (2.21)	0.51 (2.54)	179.41 (146.55)	43.23 (21.61)	43.32 (23.83)	43.11 (19.03)
		PM	15:38 (00:34)	14:52	16:26	6.57 (18.59)	−0.29 (1.35)				265.23 (234.49)			
	2nd‐to‐last	AM	9:52 (00:40)	9:00	10:55	6.37 (14.80)	−0.39 (1.52)	0.08 (1.85)	0.41 (1.8)	−0.27 (1.87)	198.59 (198.79)	48.96 (34.60)	46.24 (34.47)	51.41 (35.43)
		PM	15:28 (00:34)	14:34	16:15	8.06 (26.17)	−0.33 (1.80)				229.66 (222.79)			
2	1st	AM	9:44 (00:39)	8:50	10:50	4.19 (6.36)	−0.33 (1.68)	0.46 (1.65)	0.67 (1.74)	0.19 (1.53)	185.19 (161.63)	61.45 (44.98)	56.83 (31.35)	66.07 (56.14)
		PM	15:36 (00:33)	14:40	16:26	9.18 (23.83)	−0.01 (1.29)				261.63 (246.86)			
	2nd‐to‐last	AM	9:45 (00:38)	8:48	10:30	3.35 (10.38)	−0.84 (1.78)	0.33 (3.15)	0.85 (3.45)	−0.31 (2.68)	276.71 (229.03)	67.13 (47.32)	52.22 (27.61)	86.64 (60.56)
		PM	15:44 (00:33)	14:40	16:25	24.52 (42.27)	−0.44 (3.07)				230.62 (227.07)			
4	1st	AM	9:50 (00:37)	9:19	10:26	7.75 (17.55)	−0.66 (3.71)	0.53 (2.28)	0.84 (2.50)	0.13 (1.97)	191.91 (153.11)	48.78 (30.13)	44.05 (27.07)	54.06 (33.25)
		PM	15:40 (00:33)	14:40	16:19	19.37 (41.84)	−0.07 (2.29)				203.05 (181.28)			
	2nd‐to‐last	AM	9:51 (00:37)	9:02	10:35	3.25 (7.07)	−0.55 (1.44)	0.37 (1.75)	1.07 (1.63)	−0.49 (1.53)	204.03 (194.65)	60.41 (43.82)	51.3 (38.83)	72.56 (48.15)
		PM	15:45 (00:33)	15:0	16:15	8.08 (22.85)	−0.19 (1.39)				287.11 (227.97)			
8	1st	AM	9:55 (00:37)	9:03	10:56	10.31 (25.26)	−0.27 (1.91)	0.52 (2.14)	−0.15 (1.41)	1.08 (2.49)	190.05 (183.51)	48.28 (41.14)	41.77 (42.61)	55.18 (39.61)
		PM	15:31 (00:33)	14:38	16:19	13.53 (26.81)	0.25 (1.08)				205.41 (176.61)			
	2nd‐to‐last	AM	9:50 (00:37)	8:55	10:38	3.26 (7.80)	−0.56 (1.78)	0.64 (1.71)	0.80 (2.19)	0.48 (1.01)	183.24 (176.07)	50.27 (34.05)	59.66 (39.27)	39.55 (23.46)
		PM	15:27 (00:33)	14:30	16:40	5.03 (7.32)	0.04 (1.04)				254.15 (187.78)			
12	1st	AM	10:03 (00:37)	9:31	10:57	10.28 (21.33)	−0.05 (1.60)	0.40 (1.73)	0.68 (1.59)	0.04 (1.88)	154.16 (165.30)	41.06 (39.10)	42.44 (41.73)	38.79 (35.74)
		PM	15:46 (00:33)	15:0	16:32	14.21 (26.29)	0.31 (1.18)				173.19 (176.61)			
	2nd‐to‐last	AM	9:53 (00:37)	9:04	10:48	8.26 (21.62)	−0.13 (1.83)	0.52 (1.96)	0.69 (2.32)	0.19 (0.92)	152.71 (138.94)	45.64 (38.29)	51.28 (43.29)	35.48 (25.32)
		PM	15:51 (00:33)	15:0	16:55	15.08 (29.93)	0.43 (1.03)				212.46 (176.37)			

#### 2.3.1 Saliva Collection

For saliva collection, the current study used SalivaBio Children's Swab (Salimetrics item no.: 5001.06), designed for children from 6 months to 6 years of age. This method provides clean saliva samples and increases adherence to collection protocol compared to passive drooling (Rohleder and Nater 2009). Research assistants place the swab in the mouths (under the tongue, if possible) of children for 1–3 min, depending on the saturation progress of the swab. Some participants took more than 3 min to finish. To minimise the stimulation of salivary flow, children were instructed not to chew on the swab. After collection, the samples were preserved and transported on ice to the research base, temporarily kept at −20°C. The samples were subsequently packed with dry ice and transported to the laboratory in another city overnight by flight, where they were kept at −80°C until being assayed. Variations in cortisol and sAA are subject to many other factors, such as food intake, physical activities, and sleep.

All samples were collected one‐on‐one by trained research assistants in a place with some privacy near or inside the classrooms. Instructions were given regarding food intake, sleep, and physical activities. Specifically, the assistants were required to ensure that the children (1) did not eat, drink or sleep for at least half an hour before sampling (Hanrahan et al. 2006; Lumian et al. 2016); (2) rinsed their mouths at least 10 min before sampling, if they did not do so after eating food (Yang et al. 2017); (3) did not have meals scheduled within 1 h after sampling (to avoid anticipatory surges, as suggested by Watamura et al. 2010); and (4) completely cooled down and remained physically inactive for at least 15 min before sampling (Yang et al. 2017). Violations of the instructions and medication intake (e.g., cold medicine; Granger et al. 2009) were documented accordingly.

#### 2.3.2 Measures

##### 2.3.2.1 Cortisol

The concentration of cortisol was assayed through the enzyme‐linked immunosorbent assay (ELISA) method using the Human Cortisol Elisa Kit provided by Cusabio (catalogue no.: CSB‐E05111h), which has been used in previous human research (e.g., G. Chen et al. 2018). The sensitivity range of the kits was 0.0490 ng/mL to 200 ng/mL. Whenever possible, all samples from a single participant were assayed in duplicate—on the same assay plate to minimise the inter‐assay variation. Any pair of samples with variation greater than 10% was re‐assayed later. Samples with concentrations higher than the upper sensitivity range were diluted and re‐assayed. The intra‐ and inter‐assay coefficients of the variant were 5.08% and 14.54%, respectively.

##### 2.3.2.2 Salivary Enzyme *α*‐amylase (sAA)

The concentration of sAA was assayed using the enzyme‐linked immunosorbent assay (ELISA) method with the Human alpha‐amylase (AMY1) ELISA Kit provided by Cusabio (catalogue no.: CSB‐E14075h). The ELISA method was theoretically superior to enzyme‐kinetic methods because they assess sAA concentration precisely instead of using sAA activity as a proxy (Rohleder and Nater 2009). The sensitivity range of the kits was 7.8–500 IU/mL. Again, samples were assayed in duplicate, following the same principles as cortisol. The intra‐ and inter‐assay coefficients of the variant were 5.29% and 5.18%, respectively.

##### 2.3.2.3 Behavioural Problems

Before children entered preschool and at the end of the 12 weeks, primary caregivers completed the externalising and internalising behaviour subscales of the Social Skills Improvement System‐Rating Scales (*SSIS‐RS*; Gresham and Elliott 2008) on a four‐point Likert scale (0 = *never*, 1 = *seldom*, 2 = *often*, and 3 = *almost always*). The Chinese version of the *SSIS–RS* parent form showed strong psychometric properties (Cheung, Siu, and Brown 2017). In this study, Cronbach's *α*s of internalising and externalising subscales were 0.69 and 0.78 (pre‐entry) and 0.85 and 0.87 (Week 12), respectively.

##### 2.3.2.4 School Refusal

At the end of the 12 weeks, primary caregivers completed two sub‐scales of the School Refusal Assessment Scale‐Revised (SRAS‐R; Kearney, Chapman, and Cook 2005), that is, (A) avoiding school‐related stimuli that provoke a negative feeling and (B) avoiding school to gain attention from caregivers, using a 7‐point Likert‐type scale from *never* (0) to *always* (6). Each sub‐scale contains six items on the frequency of specific refusal behaviour for the entire 12‐week period. The scale has been successfully used among parents of 4‐year‐olds with acceptable reliability and concurrent validity (Geddes, Murrell, and Bauguss 2010). In this study, the Cronbach's *α*s of Sub‐scales (A) and (B) were 0.71 and 0.72, respectively.

##### 2.3.2.5 Control Variables

Within‐person variables included linear time (no. of days since entry into preschool), quadratic time (linear time^2), sampling time point (in minutes since the start of the day, or 0:00 AM), and medication usage (1 = yes, 0 = no; yes if the children have taken oral medications within 12 h before saliva collection). The linear and quartic time factors were included to capture the trajectory of change in dependent variables over time. Variations in sampling time and medication usage were common sources of noise in assessing these biological markers following a diurnal pattern (e.g., Albers et al. 2016; Granger et al. 2009). No cubic time factor was included because the duration was shorter, and the participants were older in this study than in Albers et al. (2016). Between‐person level variables included child age (in months), sex (1 = male, 0 = female), family SES, left‐behind status (1 = yes, 0 = no; yes if at least one of the parents was working elsewhere for at least 6 months in the past year and thus left their children behind in their hometown) and ethnic minority status (1 = yes, 0 = no). Family SES was represented by a composite score consisting of six standardised factors, including father's education, mother's education, father's occupation, mother's occupation, father's income and mother's income (see also Cohen, Doyle, and Baum 2006). These individual and family contextual factors are associated with other stressors in children's life such as poverty (Eckstein‐Madry, Piskernik, and Ahnert 2021), lack of emotional support from parents (Vermeer and van IJzendoorn 2006), and how capable they were in dealing with stress (e.g., age), therefore informing children's stress response to boarding.

#### 2.3.3 Data Preparation

Log‐10 transformation was applied to raw cortisol concentration to exclude outliers and reduce variance and deviation from normality (see also Albers et al. 2016; Bernard et al. 2015). After the transformation, the *change in cortisol from morning to afternoon* (LDC) was calculated by the formula: mid‐afternoon − mid‐morning cortisol. The daily sAA corresponds to the *area under the curve with respect to ground* (AUC) for each day, based on the formula suggested by Pruessner et al. (2003) (i.e., (mid‐morning sAA + mid‐afternoon sAA) × time duration between sampling/2).

### 2.4 Data Analysis

The descriptive statistics of cortisol and sAA concentrations with collection time and correlations among variables were first generated using SPSS24. Then, *t*‐tests and chi‐square tests were conducted to compare boarders and non‐boarders to detect any potential systematic differences between them. Further analysis was conducted to test the hypotheses using mixed‐effects modelling, as by Albers et al. (2016). Mixed‐effects modelling was conducted using Mplus7 since it allows the pattern across time for dependent variables to vary for each individual instead of estimating the pattern at the group level, and it enables convenient examination of inter‐individual predictors for the variabilities in patterns. Of a possible 1342 samples in total, 991 (73.8%) and 900 (67.1%) samples were included in the final analysis for cortisol and sAA, respectively. Missing data were due to drop‐out (2.7%), unavailability for collection (e.g., absent, tardy; 14.0%), uncooperativeness (3.7%), an insufficient amount collected (cortisol: 4.8%; sAA: 11.8%), and outliers (i.e., more than three standard deviations above the mean) (cortisol: 1.0%; sAA: 0.7%). Values for each pair of samples were averaged to get the concentration. Given that deviation from normality was presented for some variables (e.g., SES in Table 5), the missing values were estimated using full information maximum likelihood estimation (FIML) with nonnormality robust standard errors. All models had acceptable model fits (refer to Table 6 for details).

**TABLE 5:** Group differences between boarders and non‐boarders and correlations among the variables.

	Boarders (1)	Non‐boarders (−1)	Test statistics																		
	*n* = 129	*n* = 111																			
	*M* (SD)	*M* (SD)	*t*/*χ*2	1	2	3	4	5	6	7	8	9	10	11	12	13	14	15	16	17	18
1. Time linear (in days)	N/A	N/A	N/A	—																	
2. Time quadratic	N/A	N/A	N/A	0.97***	—																
3. Age (in months)	48.4 (9.4)	39.4 (8.1)	4.00***	N/A	N/A	—															
4. Sex (1 = male, 0 = female)	N/A	N/A	0.42	N/A	N/A	−0.11	—														
5. SES	−0.4 (0.3)	0.3 (0.8)	−4.44***	N/A	N/A	−0.38**	0.27*	—													
6. Left‐behind status (1 = yes, 0 = no)	N/A	N/A	1.64	N/A	N/A	−0.13	0.05	0.21	—												
7. Ethnic minority status (1 = yes, 0 = no)	N/A	N/A	2.43	N/A	N/A	−0.11	0.33**	0.41**	0.19	—											
8. Medication (1 = yes, 0 = no)	N/A	N/A	0.50	0.08*	0.04	0.04	−0.05	0.04	−0.04	0.06	—										
9. AM sampling time	9:49 (0:29)	9:58 (0:25)	−3.69***	0.04	0.06	−0.24***	−0.01	0.17***	−0.03	0.04	0.01	—									
10. PM sampling time	15:43 (0:29)	15:34 (0:28)	3.69***	0.07	0.09*	−0.05	−0.16***	−0.06	−0.08	−0.04	0.07	0.37***	—								
11. LDC of cortisol	0.6 (2.1)	0.16 (1.9)	2.30*	0.05	0.04	0.03	−0.01	−0.06	−0.04	0.07	0.01	−0.03	0.12**	—							
12. AUC of sAA	49.1 (36.0)	53.4 (40.2)	−1.12	−0.09	−0.1*	−0.12*	−0.05	0.06	−0.09	−0.06	0.12*	0.03	0.08	−0.08	—						
13. Internalising behaviour (pre‐entry)	15.6 (2.9)	15.7 (3.6)	−0.09	N/A	N/A	0.23	0.12	−0.15	−0.27*	0.05	0.01	−0.05	−0.05	0.04	−0.02	—					
14. Externalising behaviour (pre‐entry)	20.1 (4.1)	19.4 (4.4)	0.65	N/A	N/A	0.20	0.01	−0.08	−0.17	0.10	0.00	−0.11*	0.03	0.10*	−0.03	0.66***	—				
15. Internalising behaviour (w12)	16.4 (4.5)	15.3 (4.3)	0.91	N/A	N/A	0.13	−0.13	−0.16	−0.26	−0.17	0.07	−0.06	−0.05	0.03	0.14*	0.41**	0.16	—			
16. Externalising behaviour (w12)	20.3 (6.0)	19.4 (5.5)	0.55	N/A	N/A	0.02	−0.25	−0.09	−0.24	−0.25	0.06	−0.01	0.03	0.04	0.15**	0.31*	0.36**	0.78***	—		
17. School refusal (A)	10.2 (7.0)	7.5 (5.1)	1.58	N/A	N/A	−0.03	0.08	−0.15	−0.27	0.04	0.02	−0.10*	−0.02	0.11*	0.10	0.23	0.30*	0.45**	0.44**	—	
18. School refusal (B)	18.1 (7.7)	13.0 (6.0)	2.61*	N/A	N/A	0.08	0.05	−0.13	−0.18	−0.05	0.00	−0.17***	−0.07	0.07	0.05	0.10	0.20	0.30*	0.27	0.74***	—
			Mean	22.4	922.9	44.0	N/A	0.0	N/A	N/A	N/A	9:53	15:39	0.4	51.0	15.6	19.8	15.9	19.9	8.9	15.8
			SD	20.6	1235.1	9.8	N/A	0.7	N/A	N/A	N/A	00:27	00:29	2.0	38.0	3.2	4.2	4.4	5.8	6.3	7.4
			Skewness	0.66	1.15	0.32	N/A	1.95	N/A	N/A	N/A	0.31	−0.03	0.97	1.42	0.30	0.28	0.51	0.40	0.43	0.16
			Kurtosis	−1.07	−0.23	−0.93	N/A	4.18	N/A	N/A	N/A	1.04	−0.63	8.30	2.31	−0.41	−1.12	−0.78	−1.04	−0.61	0.34

*Note: N*s = 392–671 for Time linear, Time quadratic, Medication, AM/PM Sampling time, LDC of cortisol and AUC of sAA; *N*s = 51–61 for the other variables. Time linear and Time quadratic were the same for boarders and non‐boarders because the sampling days were uniform.

Abbreviations: AUC = area under the curve with respect to ground of saliva alpha‐amylase, LDC = change in cortisol from morning to afternoon, *M* = mean, School refusal (A) = avoiding school‐related stimuli that provoke a negative feeling, School refusal (B) = avoiding school to gain attention from caregivers, SD = standard deviation, w12 = week 12.

* *p* < 0.05.

** *p* < 0.01.

*** *p* < 0.001, two‐tailed.

**TABLE 6:** Model fit indexes.

Model	Description	*χ* ^2^/*df*	CFI	TLI	RMSEA	SRMR	
						Within	Between
# 1.1	Cortisol‐intercept only	N/A^a^	1.00	1.00	0.00	0.00	0.00
# 1.2	sAA—Intercept only	N/A^a^	1.00	1.00	0.00	0.00	0.00
# 2.1	Cortisol‐level 1 (within‐person)	N/A^a^	1.00	1.00	0.00	0.00	0.00
# 2.2	sAA—Level 1 (within‐person)	N/A^a^	1.00	1.00	0.00	0.00	0.00
# 3.1	Cortisol‐boarding (all days)	N/A^a^	1.00	1.00	0.00	0.00	0.00
# 3.1.1	Cortisol‐boarding (1st weekday)	N/A^a^	1.00	1.00	0.00	0.00	0.00
# 3.1.2	Cortisol‐boarding (2nd to last weekday)	N/A^a^	1.00	1.00	0.00	0.00	0.00
# 3.2	sAA‐boarding (all days)	N/A^a^	1.00	1.00	0.00	0.00	0.00
# 3.2.1	sAA‐boarding (1st weekday)	N/A^a^	1.00	1.00	0.00	0.00	0.00
# 3.2.2	sAA‐boarding (2nd to last weekday)	N/A^a^	1.00	1.00	0.00	0.00	0.01
# 4.1	Model 3.1.2 control variable analysis	N/A^a^	1.00	1.00	0.00	0.00	0.01
# 4.2	Model 3.2.2 control variable analysis	N/A^a^	1.00	1.00	0.00	0.00	0.00
# 4.3	Regression‐internalising behaviour	N/A^a^	1.00	1.00	0.00	N/A	0.00
# 4.4	Regression‐externalising behaviour	N/A^a^	1.00	1.00	0.00	N/A	0.00
# 4.5	Regression‐school refusal (A)	N/A^a^	1.00	1.00	0.00	N/A	0.00
# 4.6	Regression‐school refusal (B)	N/A^a^	1.00	1.00	0.00	N/A	0.00
# 5.1	Cortisol‐all free	N/A^a^	1.00	1.00	0.00	0.00	0.00
# 5.1.1	Cortisol‐all constrained	1.79	1.00	0.99	0.05	0.03	0.00
# 5.2	sAA‐all free	N/A^a^	1.00	1.00	0.00	0.00	0.00
# 5.2.1	sAA‐all constrained	7.15	0.99	0.92	0.14	0.05	0.00
# 5.2.2	Constrained time Linear → AUC	1.23	1.00	1.00	0.03	0.00	0.00
# 5.2.3	Constrained time Quadratic → AUC	0.02	1.00	1.01	0.00	0.00	0.00

^a^ *df* = 0, *χ* ^2^ */df* cannot be calculated. All models were evaluated by the ratio of the chi‐square test to degrees of freedom (*χ* ^2^/*df*), the root‐mean‐square error of approximation (RMSEA), the Tucker‐Lewis index (TLI), the comparative fitting index (CFI), and the standardised root mean square residual (SRMR). Criteria for ideal model fit would be *χ* ^2^/*df* < 3, CFI/TLI > 0.95 and RMSEA/SRMR < 0.08 (Hu and Bentler 1999), and model fits of all models mentioned in the Results section were presented.

The LDC and AUC of boarders and non‐boarders were compared with separate models longitudinally upon the children entering preschools. An intercept‐only model was first examined to assess the proportion between‐ and within‐person variance, controlling for several within‐person factors. To test H1‐1 and H1‐2, boarding status was added as a between‐person predictor for LDC and AUC. Analysis was conducted first for all days together and then separately for the first and second‐to‐last weekdays. On the second‐to‐last weekdays, boarders had already stayed at preschools for the past few consecutive days, which may present a better opportunity to detect group differences. To test H2‐1 and H2‐2, multi‐group comparisons were conducted to examine the moderating effect of boarding status on the associations between time factors (linear time and quadratic time) and outcome variables (LDC and AUC). Models with the path coefficients (linear time/quadratic time → LDC/AUC) constrained to be equal between the boarding and non‐boarding groups were compared to models with all path coefficients freely estimated, using the Satorra–Bentler scaled chi‐square tests (Satorra and Bentler 2001). If the overall between‐group invariance were not established, the invariance of path coefficients would be tested individually. To test H3, school refusal and behavioural problems were regressed on boarding status, controlling for between‐person covariates.

## 3 Results

The results section consists of four parts: the first part includes descriptive statistics and *t*‐tests and chi‐square tests examining the difference between boarders and non‐boarders on dependent variables and covariates, as well as an inspection of the intercept‐only model for the mixed‐effects models. Second, boarding was included at the between level as a predictor for average LDC and daily sAA. Third, boarding status was examined as a predictor for school refusal sub‐scales in multiple regressions. Fourth, multi‐group analyses were conducted to explore the differences between boarders and non‐boardings in trajectories for LDC and daily sAA over time.

The descriptive statistics of cortisol and sAA are shown in Table 4. For group comparisons and correlations among variables, please refer to Table 5. Boarders were older (*t*(59) = 4.00, *p* < 0.001) and lower in family SES (*t*(37) = −4.45, *p* < 0.001). Boarders experienced a larger rise in cortisol than non‐boarders over the day (LDC), *t*(455) = 2.30, *p* = 0.022, but not a higher total AUC of sAA, *t*(390) = −1.12, *p* = 0.263, which implies a higher level of stress for boarders compared to that for non‐boarders. Boarders exhibited more school refusal behaviour to gain attention from caregivers (School refusal [B]) at Week 12, *t*(50) = 2.61, *p* = 0.012.

The intercept‐only model indicated a significant variance of LDC and AUC both at the within‐person and between‐person levels (variance of between‐person LDC marginally significant, *p* = 0.052). The intraclass correlation coefficients (ICCs) were 7% and 37% for LDC and AUC, respectively. Thus, the multi‐level model separating within‐person and between‐person variance was appropriate. In addition, the intercept of LDC was significantly larger than zero, *β* = 0.41, *p* = 0.001. More specifically, the intercept of boarders was significant, *β* = 0.61, *p* < 0.001, whereas non‐boarders' intercept was insignificant. The significant intercept indicated a significant morning‐to‐afternoon rise in cortisol during the 12 weeks for boarders only.

### 3.1 Boarding and Average Cortisol Rise and Daily sAA

Boarding did not significantly predict LDC or AUC when all days were considered. The effect of boarding was then tested using the sub‐sets of data on the first and the second‐to‐last weekdays. Boarding had no significant effect on AUC. Nevertheless, boarding significantly predicted LDC on the second‐to‐last weekdays (*β* = 0.76, *p* < 0.001) but not on the first weekdays. Thus, boarders had a larger morning‐to‐afternoon rise in cortisol than non‐boarders after a few consecutive days of staying at school, indicating that they experienced elevated stress compared to non‐boarders in the later days of the week. After controlling for child age, sex, family SES, left‐behind status, and ethnic minority status, the effect of boarding on LDC on the second‐to‐last weekdays remained significant (*β* = 0.64 *p* = 0.023). The model explained 68% of the between‐person variance in LDC as indicated by *R*‐squared. For details, refer to Table 7.

**TABLE 7:** Results of the regression analyses on the association among boarding, stress responses and social adjustment.

	Model 4.1		Model 4.2		Model 4.3		Model 4.4		Model 4.5		Model 4.6	
	LDC of cortisol		AUC of sAA		Internalising (w12)		Externalising (w12)		School refusal (A)		School refusal (B)	
	*β* (S.E.)	*p*	*β* (S.E.)	*p*	*β* (S.E.)	*p*	*β* (S.E.)	*p*	*β* (S.E.)	*p*	*β* (S.E.)	*p*
Intercept	−18.77** (6.88)	0.006	1.67 (3.42)	0.624	1.90* (0.77)	0.014	2.61** (0.76)	0.001	2.64** (0.77)	0.001	3.02*** (0.69)	< 0.001
Within‐person												
Time linear	0.37 (0.30)	0.222	0.14 (0.27)	0.592	—	—	—	—	—	—	—	—
Time quadratic	−0.34 (0.32)	0.283	−0.32 (0.26)	0.211	—	—	—	—	—	—	—	—
AM sampling time	0.10 (0.11)	0.359	0.03 (0.11)	0.777	—	—	—	—	—	—	—	—
PM sampling time	0.11 (0.09)	0.208	0.01 (0.10)	0.889	—	—	—	—	—	—	—	—
Medication (1 = yes, 0 = no)	0.05 (0.03)	0.136	−0.06 (0.08)	0.441	—	—	—	—	—	—	—	—
Between‐person												
Boarding status (1 = boarder, −1 = non‐boarder)	0.64* (0.28)	0.023	0.09 (0.19)	0.645	0.03 (0.15)	0.850	0.00 (0.17)	0.979	0.29* (0.15)	0.043	0.45*** (0.11)	< 0.001
Age (in months)	0.02 (0.33)	0.960	−0.15 (0.18)	0.422	0.01 (0.13)	0.918	−0.06 (0.13)	0.634	−0.16 (0.11)	0.130	0.01 (0.10)	0.916
Sex (1 = male, 0 = female)	0.20 (0.26)	0.442	−0.07 (0.18)	0.693	−0.14 (0.13)	0.272	−0.20 (0.12)	0.087	−0.29 (0.17)	0.090	−0.20 (0.15)	0.169
SES	−0.19 (0.20)	0.340	0.05 (0.16)	0.776	0.02 (0.09)	0.871	0.08 (0.12)	0.520	0.08 (0.14)	0.559	0.06 (0.14)	0.673
Left‐behind status (1 = yes, 0 = no)	−0.24 (0.15)	0.113	−0.19 (0.14)	0.177	−0.13 (0.12)	0.283	−0.17 (0.12)	0.150	−0.26* (0.11)	0.013	−0.15 (0.10)	0.152
Ethnic minority status (1 = yes, 0 = no)	0.06 (0.32)	0.844	0.01 (0.21)	0.967	−0.13 (0.14)	0.355	−0.23 (0.13)	0.066	0.14 (0.14)	0.334	0.01 (0.13)	0.933
Internalising behaviour (pre‐entry)	—	—	—	—	0.42*** (0.12)	< 0.001	—	—	—	—	—	—
Externalising behaviour (pre‐entry)	—	—	—	—	—	—	0.37*** (0.10)	< 0.001	—	—	—	—

*Note: N*s = 305 for Models 4.1 and 4.2, *N*s = 61 for Models 4.3–4.6.

Abbreviations: *β* = standardised path coefficients, AUC = area under the curve with respect to ground of saliva alpha‐amylase, LDC = change in cortisol from morning to afternoon, S.E. = standard error, School refusal (A) = avoiding school‐related stimuli that provoke a negative feeling, School refusal (B) = avoiding school to gain attention from caregivers, w12 = week 12.

* *p* < 0.05.

** *p* < 0.01.

*** *p* < 0.001, two‐tailed.

### 3.2 Boarding and Social Adjustment

Multiple regressions on social adjustment indicators (models 4.3–4.6) showed that boarding significantly predicted school refusal (B), *seeking caregivers' attention*, *β* = 0.45, *p* < 0.001, after controlling for demographics. Boarding also predicted school refusal (A), *avoiding school‐related stimuli provoking negative affectivity*, *β* = 0.29, *p* = 0.043. Thus, boarders displayed higher school refusal behaviour due to *seeking caregivers' attention* (B) and *avoiding school‐related stimuli provoking negative affectivity* (A) than non‐boarders. After accounting for baseline behavioural problems and demographics, boarding was not significantly associated with externalising (*β* = 0.00, *p* = 0.979) or internalising (*β* = 0.03, *p* = 0.850) behaviour. The details of the abovementioned models are presented in Table 7. The models explained 26%, 28%, 21%, and 19% of the variance in internalising behaviour, externalising behaviour, school refusal (A), and school refusal (B), respectively, as indicated by the *R* ^2^.

### 3.3 Boarding and Changes in Cortisol Rise and Daily sAA Over Time

Multi‐group analysis compared the change trajectories over time in LDC and AUC for boarders and non‐boarders. The constrained and unconstrained models were invariant for LDC of cortisol, ∆*χ* ^2^_SB_ (2) = 3.57, *p* = 0.167, but not for AUC of sAA, ∆*χ* ^2^_SB_ (2) = 14.31, *p* < 0.001. Whereas quadratic time significantly predicted the AUC of sAA for the non‐boarding group (*β* = −0.50, *p* = 0.018), the relationship was not significant for the boarding group (*β* = −0.11, *p* = 0.709), ∆*χ* ^2^_SB_ (1) = 13.86, *p* < 0.001. Moreover, although a significant inter‐group difference was noted for the path *linear time* → AUC, ∆*χ* ^2^_SB_ (1) = 12.83, *p* < 0.001, the prediction effect of linear time was insignificant in both groups. Whereas for non‐boarders, the daily sAA level decreased over the 12 weeks at an accelerating rate, no significant relationships between time and sAA were noted for boarders. The results suggested that based on sAA, the non‐boarding group displayed a decreasing trend in stress over the 12 weeks, while no such trend was noted for the boarding group. For boarders, within‐person variances explained for LDC and AUC were 6% and 2%, respectively; for non‐boarders, within‐person variances explained for LDC and AUC were 1% and 11%, respectively (for details of model tests, see Table 8).

**TABLE 8:** Multi‐group comparison of time factors predicting LDC and AUC.

Fixed effect	Boarding group			Non‐boarding group			Test for invariance	
	*β*	S.E.	*p*	*β*	S.E.	*p*	∆*χ* ^2^_sb_	*p*
On LDC of cortisol							3.57	0.170
Within‐person								
Time linear	0.32	0.33	0.328	0.30	0.31	0.337	N/A	N/A
Time quadratic	−0.32	0.35	0.352	−0.24	0.28	0.395	N/A	N/A
AM sampling time	−0.14***	0.04	< 0.001	0.08	0.11	0.507		
PM sampling time	0.26**	0.09	0.006	0.00	0.09	0.975		
Medication (1 = yes, 0 = no)	0.01	0.05	0.807	−0.01	0.04	0.863		
On AUC of sAA							14.31***	< 0.001
Within‐person								
Time linear	0.13	0.33	0.702	0.17	0.22	0.437	12.83***	< 0.001
Time quadratic	−0.11	0.30	0.709	−0.50*	0.21	0.018	13.86***	< 0.001
AM sampling time	−0.10	0.09	0.257	−0.02	0.09	0.820		
PM sampling time	0.05	0.09	0.538	0.05	0.11	0.670		
Medication (1 = yes, 0 = no)	0.12	0.09	0.205	0.04	0.07	0.539		

*Note: N*s = 671. *β* = standardised path coefficients. The coefficients reported were based on the unconstrained models (Models 6.1 and 6.2). *χ* ^2^_sb_ for the entire model was generated by comparing the all‐free models with the all‐constrained models (Models 5.1.1 and 5.2.1). *χ* ^2^_sb_ for individual paths were generated by comparing the models with each corresponding path constrained to be equal across groups (Models 5.2.2 and 5.2.3) with the all‐constrained model (Model 5.2.1).

* *p* < 0.05.

** *p* < 0.01, two‐tailed.

*** *p* < 0.001.

In summary, boarding only positively predicted the change in cortisol from morning to afternoon (LDC) on the second‐to‐last weekdays but not daily sAA, so H‐1 was partially supported, while H‐2 was not. Multi‐group analysis comparing the change trajectory of LDC and daily sAA found a significant difference between boarders and non‐boarders only for daily sAA but not LDC. Thus, H2‐2 was supported, but H2‐1 was not. H3 was also partially supported since we found that boarders, compared to non‐boarders, displayed more school refusal behaviours but not externalising or internalising behaviours.

## 4 Discussion

The current study examined the effect of an extreme case of long‐hour childcare, namely, boarding, on rural Chinese children's stress responses and social adjustment over 12 weeks upon preschool entry. The results showed that boarders experienced a larger morning‐to‐afternoon rise in cortisol than non‐boarders on the second‐to‐last weekdays. Whereas non‐boarders experienced an accelerated decline in daily sAA level across time, boarders did not experience such a reduction. Finally, boarders exhibited more school refusal behaviour than non‐boarders, although no group differences were noted for behavioural problems.

### 4.1 Association Among Boarding, Diurnal Cortisol Levels and Trajectories

This study is the first to examine the differences in stress responses between boarders and non‐boarders. Past evidence showed that more hours spent in childcare predicted a larger morning‐to‐afternoon rise in cortisol in childcare (Drugli et al. 2017; Lumian et al. 2016). Consistent with the literature that children under stress display a morning‐to‐afternoon rise in cortisol (Drugli et al. 2017; Lumian et al. 2016), we found that boarders who spent more time at preschools experienced a larger morning‐to‐afternoon rise in cortisol than non‐boarders. Drugli et al. suggested a dose‐response relationship between hours in childcare and a rise in cortisol over the day, mainly due to tiredness after long periods spent interacting with caregivers and peers.

Interestingly, the difference in cortisol rise between boarders and non‐boarders was noted only on the second‐to‐last weekdays but not on the first weekdays. On the one hand, considering the decreasing or flattened diurnal pattern of cortisol on days at home (e.g., Tervahartiala et al. 2021; Watamura et al. 2010), the common experience of spending weekends at home among boarders and non‐boarders might somehow ‘reset’ the boarding effect in the past week and bring boarders and non‐boarders to an equal level of cortisol. This ‘reset’ could likely be supported by Gunnar et al.'s (1981) finding that it took about one day for elevated cortisol levels to recover following maternal separation. In addition, during weekends, boarders could access parental support, which is critical in restoring the emotional equilibrium of young children (Ahnert and Lamb 2003). On the other hand, there may be a carry‐over effect of time on the diurnal cortisol pattern when time spent at school is prolonged (Lumian et al. 2016). Because boarders could not access emotional comfort from their primary caregivers during the weekdays, their stress was not effectively dealt with and might have accumulated over the weekdays. Thus, it is reasonable that group differences in cortisol rise between boarders and non‐boarders became detectable only after a few days. Another important fact about the carry‐over effect was that boarders stayed overnight at school. Higher hypothalamic‐pituitary‐adrenal axis activity overnight may suppress morning cortisol levels, producing a flattened or rising diurnal pattern (Bernard et al. 2015). Because boarders must sleep in large communal dormitories during weekdays, they may be exposed to sleep interruptions from other boarders and thus experience elevated levels of stress overnight (e.g., high hypothalamic–pituitary–adrenal axis activities of cortisol level). Unfortunately, sleep quality was not assessed in this study.

The findings on the morning‐to‐afternoon cortisol rise over time during the transition were partially consistent with past evidence (e.g., Bernard et al. 2015): the whole sample showed a morning‐to‐afternoon rise in cortisol across all the collection days. However, Bernard et al. detected an increase in the size of the rise during the 10 weeks upon entry into childcare, whereas no linear or quadratic effect of time on the rise in cortisol was found for either boarders or non‐boarders in this study. Bernard et al. explained that children rose earlier in the morning as time passed, and the time between waking up and morning sampling grew longer. The time between waking up and morning sampling may have remained relatively stable in the current study, especially for boarders, because school routines restricted their wake‐up time. Because no time effect was detected on the rise in cortisol for both groups, there was no difference in change trajectories between boarders and non‐boarders to compare.

### 4.2 Association Among Boarding, Daily sAA Levels and Change Trajectories

Contrary to our expectations, we found no difference in the average daily sAA levels between boarders and non‐boarders. Because the sAA level was more relevant to the overall emotional arousal (e.g., laughter, crying, anger, and fear; Hatfield et al. 2013), this finding indicated that boarders and non‐boarders did not differ in average emotional arousal during the period. Because the study included the boarders and non‐boarders from the same classrooms, contextual factors were well controlled.

Given the relationship between sAA level and emotional arousal, the change trajectories over time would be more interesting than the average level of the period because the difference in responses may take some time to be detectable (e.g., Yang et al. 2017). Although no past evidence on sAA levels during transition to childcare has been identified, it was noted that crying and fussing behaviour was most prevalent on the first day of separation and gradually decreased on the following days (Ahnert et al. 2004). The change in daily sAA levels of non‐boarders followed the same decreasing pattern over the 12 weeks. This trend might indicate decreased emotional arousal frequency during the day for non‐boarders (Hatfield et al. 2013).

Although boarders and non‐boarders shared the same classroom environments during the day, boarders' daily sAA levels did not decrease. As discussed above, primary caregivers provide important emotional support for young children and restore them to emotional equilibrium (Ahnert and Lamb 2003). Boarders without such comfort would more likely be affected emotionally and be reactive to emotional triggers than non‐boarders. Another explanation is that prolonged separation from primary caregivers among boarders may impair their attachment security. Insecurely attached preschoolers typically exhibit worse emotional and behavioural regulation than securely attached preschoolers (Vondra et al. 2001), which may, in turn, affect daily sAA levels.

### 4.3 Association Between Boarding and Behaviour Adjustment

This study did not identify differences between boarders and non‐boarders in internalising or externalising behaviour. The relevant evidence in the literature (e.g., Ak and Sayil 2006) was almost exclusively concerned with school‐age boarders, and circumstances for preschool may differ. The absence of a detectable group difference might have been because boarders were less deprived of emotional support than orphans (e.g., Lee et al. 2010) since they could reunite with their families during weekends. It is also possible that the group differences may take a period longer than 3 months to emerge.

Boarders exhibited more school refusal behaviour due to seeking attention from caregivers and avoiding school‐related stimuli provoking negative affectivity. These findings confirm that separation anxiety and attention‐seeking motives often drive preschool‐age children's school‐refusal behaviours (Kearney, Chapman, and Cook 2005). Emotional support from caregivers is essential for preschoolers (Ahnert and Lamb 2003), but boarders lack such support and may, therefore, be more eager for caregivers' attention. Increasing evidence suggests that high‐quality parenting could protect young children against HPA malfunctioning when risk factors are presented, such as low self‐regulation and negative emotionality (Abraham, Zagoory‐Sharon, and Feldman 2021). Without adequate emotional support, boarders may find it hard to restore emotional equilibrium after experiencing negative emotions on weekdays.

### 4.4 Contributions, Limitations, and Future Directions

The findings of the current study are both theoretically and practically important. This study represents the first endeavour to compare the stress response of boarding and non‐boarding preschoolers. The analysis revealed that boarders, compared to non‐boarders, experienced a higher stress level during the transition to preschool. Although attachment theory suggests that separation from parents can induce stress in children, past evidence mostly focuses on brief separation lasting from minutes to hours (e.g., strange situations used to study attachment; Shakiba and Raby 2023) or permanent separation, such as children in foster care (e.g., van Andel et al. 2014). In contrast, repeated prolonged separation caused by boarding has received limited research attention. The unique experiences of boarding preschoolers revealed more detailed mechanisms underlying the relation between parental separation and stress by providing support for the dose‐response relationships between childcare duration and the elevation of cortisol during the day (Drugli et al. 2017) beyond the single‐day time frame. It also suggests that the stress induced by childcare could potentially carry over throughout the weekdays despite that stress indicators follow a diurnal pattern (Lumian et al. 2016). Findings on the maintained trajectory of boarding children's sAA level suggested that boarding was associated with continued HPA activation over 3 months following entry into preschool. Such prolonged HPA activation may induce malfunctioning of stress physiology in the long run (Ali and Pruessner 2012). Future studies are needed to explore detailed mechanisms involving stress levels and stress regulation or dysregulation in the association between boarding and children's psychological and behavioural well‐being in the long run.

Practically, the findings indicate that boarders need extra emotional support to adapt to the childcare context during the transition from home to preschool; given the limited emotional support from teachers available in rural preschool settings (Sun and Li 2013), boarding preschoolers are disproportionately affected by stress and in urgent need of attention. Caregivers could consider providing additional emotional support for boarders during the weekdays through voice messages, brief video conversations, or physical visits to reduce the cumulating stress of these young boarders. Apart from supporting caregiver‐child connections, educators can consider providing more emotional comfort to boarders in the evening (e.g., hugging; Cohen et al. 2015). They can also incorporate stress‐reduction components in the evening time, such as physical activities (Martikainen et al. 2013), and make the evening and sleep environment less stressful for boarders (e.g., providing soft toys as sleeping aids; Kushnir and Sadeh 2012). Efforts at the policy level should focus on improving the quality of boarding preschools, for example, funding the preschools to reduce the child‐staff ratio in the evening (Xiao et al. 2023) and providing some personal space for each child in the dormitory (Reardon et al. 2023). Building more preschools to increase the accessibility of early childhood education is also vital, such as the One Village One Preschool programme (S. Chen et al. 2019), since it reduces travel time to and from preschools and the need for boarding services.

The current study had a few methodological limitations. First, the sample size was small (*n* = 61), and the participants were all from rural China with low SES. Thus, the generalisability of the findings to other contexts, such as boarding preschools in urban areas or other cultures, may be limited. Specifically, in urban China, boarding preschools usually serve busy, high‐income families during weekdays (Tobin, Hsueh, and Karasawa 2009). These are completely different settings compared to rural boarding preschools because they are resourceful, and we know that high care quality could potentially mitigate the additional stress brought by childcare settings (Watamura, Kryzer, and Robertson 2009). Second, the children's social adjustment was reported solely by primary caregivers, whereas problem behaviours exhibited in their preschool were not measured. The effect of boarding on children's problem behaviours may be context‐specific and only observable at school; therefore, the effect may not be adequately captured by parental reports. Third, due to the small sample size and complex nature of the analysis, we did not control for clustering. Thus, classroom‐ or preschool‐level factors might influence our results. Lastly, although training and printed protocols were provided to the research assistants, inconsistent practice may have occurred due to restrictions imposed by participating schools, such as teachers or school management.

Future studies should examine the mechanisms that underlie the impact of boarding on stress responses in preschool children, especially the mechanisms related to boarders' experience in the evening and at night, since those are where the difference in experience between boarders and non‐boarders comes from. For example, it may be valuable to measure sleep quality during weekdays or add additional assessment points early in the mornings after waking up and late in the evenings before bed. Through these additional assessments, we might be able to find mediators between boarding experience and stress responses and identify periods that are especially stressful for boarders. These are important future steps to inform the design of targeted interventions. It would also be important to look for potential buffering factors for boarders, such as positive peer relationships (Ahnert et al. 2022), high childcare quality (Hatfield et al. 2013) and supportive teacher‐child relationships (Eun McDevitt and Recchia 2022), to inform ways to assist boarders during their transition to preschools.

## 5 Conclusion

The current study extends our understanding of children's stress responses during the transition to preschool and the associations between boarding and children's adjustment. On average, boarders experienced more stress than non‐boarders, as indicated by the additional morning‐to‐afternoon rise in cortisol. Because boarders' daily sAA level did not significantly decrease over the transition period, it may be more challenging for them to adapt to preschool life than non‐boarders. Finally, boarders exhibited more school refusal behaviour than non‐boarders due to their need to seek attention from caregivers and/or avoid school‐related stimuli provoking negative affectivity. The current findings suggest that boarding is challenging for young children's well‐being and adjustment upon transition from home to preschool at both physiological levels, as indicated by stress biological makers, and behavioural levels, as indicated by school refusal behaviours. The findings also contributed to the Attachment Theory, indicating that repeated weekly prolonged separation from primary caregivers is stressful for young children, and that there is a dose‐response relationship as well as the carry‐over effect of elevated cortisol level as a biological marker of stress. Stress regulation may serve as a key mechanism in understanding the association between boarding experience and children's behavioural outcomes and well‐being. Our findings highlight the urgent need to examine, in greater depth, how boarding affects young children's psychological well‐being and to design effective interventions, especially for rural children exposed to multiple stressors, including boarding.

## Ethics Statement

The terms of this arrangement have been reviewed and approved by the University of Hong Kong, following its policy on objectivity in research. We have complied with APA ethical standards in conducting the research.

## Conflicts of Interest

The authors declare no conflicts of interest.

## Data Availability

The data that support the findings of this study are available from the corresponding author upon reasonable request.
